# Cytotoxicity of Dental Cements on Soft Tissue Associated with Dental Implants

**DOI:** 10.1155/2022/4916464

**Published:** 2022-01-20

**Authors:** Prashant Bajantri, Shobha J. Rodrigues, K. Shama Prasada, Umesh Y. Pai, Thilak Shetty, Sharon Saldanha, M. Mahesh, Puneeth Hegde, Ann Sales, Sandipan Mukherjee, Vignesh Kamath

**Affiliations:** ^1^Department of Prosthodontics, Manipal College of Dental Sciences, Mangalore, Manipal Academy of Higher Education, Manipal, Karnataka, India; ^2^Department of Cell and Molecular Biology, Manipal School of Life Sciences, Manipal Academy of Higher Education, Manipal, Karnataka, India

## Abstract

**Purpose:**

To investigate and compare the cellular host response of human gingival fibroblasts (HGF) on four currently used cements. *Methods and Material*. 5 cement pellet samples were made for each of the 4 test cements (*n* = 20). The cements used for this study were zinc phosphate, zinc oxide noneugenol (ZOE), RelyX U200, and glass ionomer cement (GIC). One commercially available cell line was used to investigate the cytotoxicity of peri-implant tissues. Direct contact cell culture testing was conducted following International Organization for Standardization (ISO) methods 10993-5 and 10993-12 (MTT assay test). Cell cultures without dental cement were considered as control. Cells were allowed to grow and confluence over 48 hours after subcultivation according to standard laboratory procedures. The cells were kept in direct contact with the cement samples for 24 hours before being subjected to analysis. All specimens were tested in triplicate to validate the results. Quantitative evaluation of cytotoxicity was done to measure cell death and inhibition of cell growth. Results were analyzed using 1-way ANOVA (*a* = 0.05) followed by Tukey B post hoc test.

**Results:**

The results of the study showed that HGF was vulnerable to the dental cement test material. GIC, zinc phosphate, ZOE, and resin cement were cytotoxic in decreasing order, respectively, and significantly reduced the cell viability after exposure to HGF (*p* < 0.001).

**Conclusions:**

Within the limitations of this in vitro cellular study, results indicated that the test dental cements were cytotoxic to HGF. The highest cytotoxicity was observed in GIC followed by zinc phosphate, ZOE, and resin cement.

## 1. Introduction

The replacement of missing teeth with endosseous implants for the rehabilitation of completely and partially edentulous patients has become the standard of care in dentistry. Prosthetic reconstruction with implants involves both cement-retained and screw-retained restorations.

Cement-retained restorations score over screw-retained on account of their ease of fabrication, better aesthetics, lower cost, and freedom of treatment planning [1, 2].

Another advantage of cement-retained prosthesis is that the restorative cement possesses shock-absorbing properties which decrease the force distribution into the alveolar bone via the implant assembly [1, 3, 4]. Lacking such shock-absorbing materials, the screw-retained prosthesis accumulates these stresses inside the implant assembly which may adversely affect their success rates [5].

One of the biggest challenges associated with cement-retained prosthesis is the difficulty in clearing the residual cement used in the luting of the prosthesis to the dental implant which may leave behind excess cement in the soft tissues around the implant [6].

^.^Studies indicate that this may occur in 8.6–14.4% of cases, with the largest incidences of residual cement being associated with subgingival margins of implant restorations [7–12].

The American Academy of Periodontology attributes excess cement around dental implants as a risk factor for peri-implant disease [13].

Peri-implant diseases are complex conditions associated with inflammatory processes that may affect the soft tissues (perimucositis) and/or hard tissues (peri-implantitis) associated with dental implants and are caused by an over-reactive immune response to a consortium of subgingival, largely anaerobic Gram-negative bacteria [14–21]. The effects of these peri-implant diseases may range from resorption of bone to complete failure of the implants [22, 23]. In a study conducted by Raval et al., it was concluded that the human gingival fibroblasts (HGF) showed greater changes in cell viability than osteoblasts exposure to different luting cements [24].

The choice of luting cement for implant prosthesis also depends on the viscosity of the cement, ease of mixing, and the operator's preference. [25] In light of the above findings, it is imperative to study the nature of the luting cement and its effect on the peri-implant tissues to avoid failure of the implant.

This study aimed to investigate and compare the cellular host response of the HGF on exposure to four different commercially available test cements.

The null hypothesis was that there will be no difference in the cellular host response of the HGF in contact with the test cement.

## 2. Methods and Material

### 2.1. Preparation of the Test Cement

The four test cements used in the study are presented in Table 1. A total of 20 samples (5 cement pellets from each of the 4 types of cement) were molded in polytetrafluoroethylene (PTFE) polymer molds of dimensions (7 × 3 × 3 mm) following mixing of the respective cement in accordance with the manufacturer's instructions in an aseptic environment.

#### 2.1.1. Preparation of Zinc Phosphate Cement

The powder was added in small increments to the liquid and mixed according to the manufacturer's instructions. Once the appropriate consistency was achieved, the cement was transferred to five polytetrafluoroethylene (PTFE) polymer molds. The setting time of cement was approximately 5–9 minutes.

#### 2.1.2. Preparation of Glass Ionomer Cement (GIC) Samples

GIC powder and liquid were mixed according to the manufacturer's recommendation. Mixing was done by folding method to maintain the gel structure and was immediately transferred to the five PTFE molds. The GIC cement setting time was approximately 24 hours.

#### 2.1.3. Preparation of Zinc Oxide Noneugenol Cement Samples

The base and catalysts were mixed on a mixing pad according to the manufacturer's recommendations and transferred to five PTFE molds. The setting time of zinc oxide noneugenol was approximately 3 minutes 30 seconds.

#### 2.1.4. Preparation of Resin Cement Samples

The required quantity of material was dispensed from the clicker dispenser of the Automix syringe. Next, it was mixed and transferred to the five PTFE molds. The setting time of RelyX^TM^ U200 cement was 30 seconds.

For all the experiments, the dimension of the mold used was 7 × 3 × 3 mm. The cement once prepared was kept at room temperature for two days, washed with phosphate buffer saline of pH 7.4, air-dried in a biosafety cabinet hood (NUAIR, USA), and used for all the experiments.

### 2.2. Evaluation of Cytotoxicity

The cytotoxicity of the four test cements was tested using in-house generated HGF by MTT assay test 3-(4,5-dimethylthiazol-2-yl)-2,5-diphenyltetrazolium bromide as published earlier [26, 27]. The HGF was cultured in DMEM containing 10% FBS. All the cell lines were cultured and maintenance was carried out using class-II biosafety cell culture hood (Nuair, USA) and CO_2_ incubator (Thermo Fisher Scientific, USA). In brief, 1 × 10 [4] HGF were cultured in a 96-well plate and exposed to the four test cements by directly exposing them to the HGF for 24 hrs at 37°C with 5% CO_2_. At the end of 24 hrs, the cement was removed and incubated with MTT (5 mg/ml, Sigma, USA) per well for 4 hrs at 37°C with 5% CO_2_. Subsequently, the supernatant was removed, and the formazan crystals were dissolved with 150 µL of DMSO (Sigma, USA). The absorbance was measured at 570 nm and 630 nm using a microplate reader (Varioskan, Thermo Fisher Scientific, USA). All the experiments are conducted in triplicates and repeated 3 times.

## 3. Results

**Figure 1:**
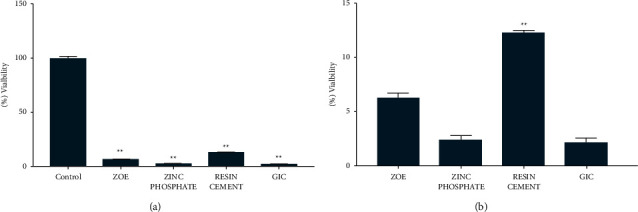
The effect of direct cement exposure on the viability of HGF cells. The cement was prepared and allowed to be set as per the manufacturer's protocol. The HGF cell viability was tested by MTT assay following 24 hours of exposure. (a) The bar graph represents the HGF viability after 24 hours of exposure along with control cells. (b) The bar graph represents the comparison of HGF viability upon exposure to four dental cements. All the experiments are performed in triplicate and repeated 3 times. ^*∗*^*P* < 0.05 was considered statistically significant.

In the present study, we tested the cytotoxic effect of 4 dental cements, namely, zinc phosphate, zinc oxide noneugenol, resin cement, and glass ionomer using MTT assay. The setting time for zinc phosphate cement, glass ionomer cement, zinc oxide noneugenol cement, and resin cement was 5 to 9 minutes, 24 hours, 3 minutes 30 seconds, and 30 seconds, respectively. The cement blocks were kept at room temperature, washed with PBS, air-dried under aseptic conditions, and directly exposed to HGF for 24 hrs. The cell viability was evaluated 24 hrs after exposure using MTT assay. The unexposed cells were kept as a control group.  Table 2 summarizes the quantitative analysis of the cytotoxicity testing after direct exposure of four test cements to HGF.  Table 3 shows the intergroup comparison of HGF mean cell count after 24-hour direct contact exposure to various dental cement materials using the post hoc Tukey test (*P* < 0.05). The HGF viability in control, zinc phosphate cement, glass ionomer cement, zinc oxide noneugenol cement, and resin cement was 99.35%, 2.46%, 2.21%, 6.6%, and 12.97%, respectively (Figure 1(a)). Resin cement showed the highest cell viability when compared with the other three cements tested (Figure 1(b)). Zinc oxide noneugenol cement also showed significantly better cell viability when compared with zinc phosphate cement and glass ionomer cement. Among the four cements tested, zinc phosphate cement and glass ionomer cement showed least cell viability.

## 4. Discussion

This study investigated and compared the cellular host response of HGF to 4 different dental types of cement. All the HGF demonstrated cytotoxic effects when exposed to the test cement. Therefore, the null hypothesis that there would be no difference in the cellular response of fibroblasts on exposure to various test cements was rejected.

The control group demonstrated significantly higher viability of gingival fibroblasts as compared to test groups. This corroborates with the study conducted by Rodriguez et al. [28], where it was found that human gingival fibroblasts were sensitive to acrylic resin, zinc oxide eugenol, and zinc phosphate cement and this exposure significantly reduced their cellular viability. In comparison of the control group with the zinc oxide eugenol group, zinc phosphate group and resin cement group, and GIC group, the viability of cells in the control group was found to be significantly higher. This suggests that the gingival fibroblasts are sensitive to cement exposure. According to Rodriguez et al. [28], both the soft tissues (fibroblasts) and the hard tissues (osteoblasts) are sensitive to commercially available luting cement. Their study also suggested that osteoblasts are comparatively less affected by the cement than fibroblasts. The findings of this study are in agreement with a study conducted by Schwap et al. and Trumpate et al. in which resin-based materials showed toxicity in cell culture tests [29, 30]. A study conducted by Stanislawski et al. [31] showed that the presence of fluoride, strontium, and aluminium ions in glass ionomer cement was lesser than the concentration needed to cause cytotoxicity to the tissues and concluded that the other major constituents of GIC are the main causative factors which contribute to its cytotoxic effect. A study conducted by Inoue et al. showed that the more the unreacted material, the higher was the toxicity of the material [32]. Sun et al. [33] tested the cytotoxicity of 3 different self-adhesive types of cement (RelyX U200, Maxcem Elite, and Multilink Speed) with and without light irradiation. The results of this study showed that cell apoptosis or necrosis rate of RelyX U200 and Maxcem Elite with light irradiation was higher than those without light irradiation. The cytotoxicity demonstrated by resin cement may be triggered by the release of monomers. Greater the content of the unreacted material, lesser would be the degree of conversion and therefore higher the cytotoxic effect [34–37].

Previous studies have shown that this reduction in cytotoxicity increases with time until no toxicity is detectable after 6 weeks [31, 38]; in a study conducted by Wilson et al. [7], it was found that in 81% of the implants that showed sulcular bleeding and/or suppuration, residual cement was present. The residual cement was then removed, and four weeks after removal of the cement in 75.7% of the cases, no signs of inflammation were noticeable. The present study however studied the effect of cytotoxicity over a 24-hour period which may be considered a limitation of this study.

## 5. Conclusion

Within the limitations of this in vitro study, the following conclusions were drawn: all the luting cements are cytotoxic to the gingival fibroblast cells; mmaximum cytotoxicity was demonstrated in GIC followed by zinc phosphate, zinc oxide noneugenol, and resin cement.

## Figures and Tables

**Table 1:** Dental cement investigated.

Cement type (groups)	Product name	Manufacturer	Setting time
Zinc phosphate	De Trey Zinc	Dentsply Sirona	5–9 minutes
Zinc oxide noneugenol	RelyX^TM^ Temp NE	3M ESPE	3 minutes 30 seconds
Resin cement	RelyX^TM^ U200	3M ESPE	24 hours
Glass ionomer	GC Gold Label	GC I	30 seconds

**Table 2:** Comparison of the viability of all the 4 experimental groups and the control group.

Groups	*N*	Mean	Std. deviation	Mean square/F statistic	*P* value
Control	4	99.353846	2.4079349	7345.647/3210.079	**<0.001**
ZOE	5	6.642051	0.4139323		
Zinc phosphate	5	2.461538	0.4501150		
Resin cement	5	12.976410	2.4410946		
GIC	5	2.219487	0.4335442		
Total	24				

**Table 3:** Intergroup comparison of HGF mean cell count after 24-hour direct contact exposure to various dental cement materials using post hoc Tukey test.

Group	Comparison with	Mean difference	Standard error	*P* value
Control	ZOE	92.7117949 ^*∗*^	1.2181156	**<0.001**
	Zinc phosphate	96.8923077 ^*∗*^	1.2206794	**<0.001**
	Resin cement	86.3774359 ^*∗∗*^	1.6252157	**<0.001**
	GIC	97.1343590 ^*∗*^	1.2194793	**<0.001**

ZOE	Zinc phosphate	4.1805128	0.2734752	≤0.001
	Resin cement	−6.3343590 ^*∗*^	1.1072744	**0.038**
	GIC	4.4225641 ^*∗*^	0.2680673	≤0.001

Zinc phosphate	Resin cement	−10.5148718 ^*∗*^	1.1100943	**0.005**
	GIC	0.2420513	0.2794867	0.995

Resin cement	GIC	10.7569 ^*∗*^	1.1087	**0.005**

HGF, fibroblast cell line; ^∗^*P* < 0.05.

## Data Availability

Data are available on request; kindly contact the corresponding author.
